# A New Insight on Stereo-Dynamics of Penning Ionization Reactions

**DOI:** 10.3389/fchem.2019.00445

**Published:** 2019-06-18

**Authors:** Stefano Falcinelli, Fernando Pirani, Pietro Candori, Brunetto G. Brunetti, James M. Farrar, Franco Vecchiocattivi

**Affiliations:** ^1^Department of Civil and Environmental Engineering, University of Perugia, Perugia, Italy; ^2^Department of Chemistry, Biology and Biotechnologies, University of Perugia, Perugia, Italy; ^3^Department of Chemistry, University of Rochester, Rochester, NY, United States

**Keywords:** Penning ionization, stereo-dynamics, metastable atoms, transition state, electron spectroscopy, crossed molecular beams

## Abstract

Recent developments in the experimental study of Penning ionization reactions are presented here to cast light on basic aspects of the stereo-dynamics of the microscopic mechanisms involved. They concern the dependence of the reaction probability on the relative orientation of the atomic and molecular orbitals of reagents and products. The focus is on collisions between metastable Ne^*^(^3^P_2, 0_) atoms with other noble gas atoms or molecules, for which play a crucial role both the inner open-shell structure of Ne^*^ and the HOMO orbitals of the partner. Their mutual orientation with respect to the intermolecular axis controls the characteristics of the intermolecular potential, which drives the collision dynamics and the reaction probability. The investigation of ionization processes of water, the prototype of hydrogenated molecules, suggested that the ground state of water ion is produced when Ne^*^ approaches H_2_O perpendicularly to its plane. Conversely, collisions addressed toward the lone pair, aligned along the water C_2v_ symmetry axis, generates electronically excited water ions. However, obtained results refer to a statistical/random orientation of the open shell ionic core of Ne^*^. Recently, the attention focused on the ionization of Kr or Xe by Ne^*^, for which we have been able to characterize the dependence on the collision energy of the branching ratio between probabilities of spin orbit resolved elementary processes. The combined analysis of measured PIES spectra suggested the occurrence of contributions from four different reaction channels, assigned to two distinct spin-orbit states of the Ne^*^(^3^P_2, 0_) reagent and two different spin-orbit states of the ionic M^+^(^2^P_3/2, 1/2_) products (M = Kr, Xe). The obtained results emphasized the reactivity change of ^3^P_0_ atoms with respect to ^3^P_2_, in producing ions in ^2^P_3/2_ and ^2^P_1/2_ sublevels, as a function of the collision energy. These findings have been assumed to arise from a critical balance of *adiabatic* and *non-adiabatic effects* that control formation and electronic rearrangement of the collision complex, respectively. From these results we are able to characterize for the first time, according to our knowledge, the state to state reaction probability for the ionization of Kr and Xe by Ne^*^ in both ^3^P_2_ and ^3^P_0_ sublevels.

## Introduction

Penning ionization is a reaction that occurs between an excited atom X^*^ and another partner M forming an excited collision complex (X···M)^*^. This complex, being immersed in the ionization continuum, spontaneously auto-ionizes

$${\text{X}}^{*}\text{ + M }\to \;{\left(\text{X}\cdot \cdot \cdot \text{M}\right)}^{*}\;\to {\left(\text{X}\cdot \cdot \cdot \text{M}\right)}^{\text{+}}\text{ + }{\text{e}}^{-}\;\to \text{ ion}\;\text{products}$$

leading to various ionic products, since in Penning ionization reactions the intermediate ionic complex (X···M)^+^ can evolve producing different final ions: M^+^ Penning ions, XM^+^ associate ions, and—in the case of molecular targets—rearrangement and dissociative ionizations are also possible (Niehaus, 1973; Brunetti and Vecchiocattivi, 1993; Siska, 1993). In particular, ionization occurs when (X···M)^*^ exhibits a lifetime, with respect to auto-ionization, shorter than the collision time, typically in the order of ~10^−12^ s. Moreover, Penning ionization, collisional auto-ionization, and chemi-ionization are often considered to be synonyms of this kind of reactions (Niehaus, 1973; Brunetti and Vecchiocattivi, 1993; Siska, 1993).

**Figure 1:**
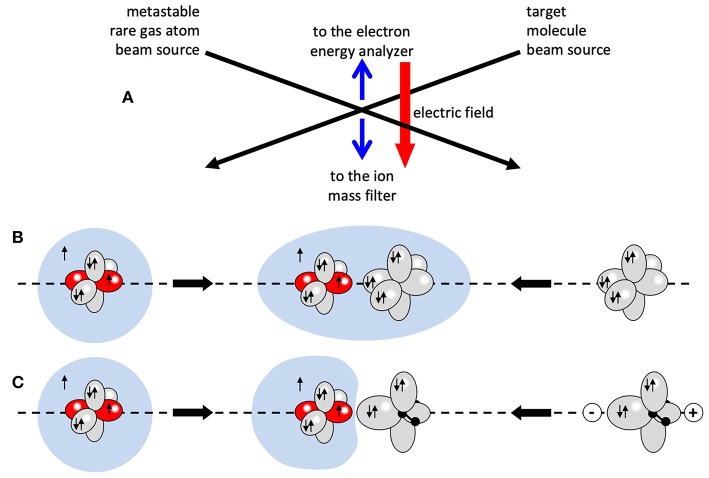
The scheme **(A)** represents a typical molecular beam study for Penning ionization: a beam of metastable rare gas atoms is crossing a beam of target molecules. Product ions and electrons are extracted from the crossing volume by an appropriate electric field. The cartoons in **(B,C)** schematically show the collision complex in the ionization of a rare gas atom and the water molecule, respectively, where H_2_O is oriented with the negative charge toward the metastable atom.

A scheme of a typical molecular beam experiment, for the investigation of Penning ionization processes under single collision condition, is reported in Figure 1A. The used setup in our experiments is a crossed beam device already outlined in previous papers (Brunetti et al., 1998, 2006). Three differentially pumped vacuum chambers during the experiment are maintained at a pressure ranging between ~10^−7^ and 10^−8^ mbar. Two chambers are used to produce a beam of metastable rare gas atoms, mainly He^*^ and Ne^*^, employing either electron bombardment and microwave discharge beam sources specially developed in our laboratory. In a third chamber the metastable atoms beam induces the single reactive collision event under study, crossing at right angle a secondary effusive beam of target particles that, in the case of the present experiments, are of Kr, Xe atoms, or of H_2_O molecules. Mass spectrometric determinations can be performed extracting product ions, coming out from the monitored auto-ionization process, by an ion optics device and filtering them by a quadrupole mass spectrometer placed below the scattering volume. A channel electron multiplier detects selected ions, recording their relative abundances. For the collection of emitted electrons and the measure of their kinetic energy content, realizing a real spectroscopy of the transition state of the studied reactions, we use a dedicated and specially designed hemispheric electrostatic analyzer located above the scattering center.

Usually, studies are carried out using metastable He^*^(2 ^1^S_0_, ^3^S_1_) with the electron configuration (e.c.) 1s 2s, Ne^*^(^3^P_2, 0_) with e.c. 2p^5^ 3s or Ar^*^(^3^P_2, 0_) atoms having e.c. 2p^5^ 3s, since they are characterized by an energy high enough to ionize most atoms or molecules and also possess a life-time longer than the collision time, even in slow collisions. The formed charged products are typically detected and analyzed in energy (the electrons) and mass (the ions) as a function of the collision energy (Niehaus, 1973; Brunetti and Vecchiocattivi, 1993; Siska, 1993).

These barrier-less reactions play an important role in biology, physics and chemistry of plasmas, planetary atmospheres, and interstellar environments (Falcinelli et al., 2015a, 2016a). They are driven by an optical potential W, defined as combination of a real, V_t_, and an imaginary part, Γ, which determine: (a) the dynamics of reactants approach and products separation, (b) the triggering of the electronic rearrangement within the transition state, respectively (Niehaus, 1973; Brunetti and Vecchiocattivi, 1993; Siska, 1993; Biondini et al., 2005a,b). More specifically, the imaginary part plays a crucial role in describing the disappearance probability of reactants, with their transformation into the final ionic products (Falcinelli et al., 2013). Accordingly, W is defined as

(1) $$W=V_{t}-\frac{i}{2}\Gamma$$

The strength of both these components is expected to vary with the center-of-mass separation (or intermolecular distance) R and with the relative orientation of two involved partners, since the spontaneous electron ejection driving the process can be strongly stereo-selective, as suggested by the cartoon in Figures 1B,C (Falcinelli et al., 2018a,b).

In previous investigations both real and imaginary components were usually considered to vary only with the separation distance, that is assuming a simple radial dependence, due to the lack of detailed information on the interaction anisotropy (related to the relative orientation of the approaching reactants and removing products), and on the stereo-selectivity of the electronic coupling within the collision complex (Gregor and Siska, 1981; Falcinelli et al., 2016b). Under such an assumption, the analysis of the experimental findings provided only semi-quantitative information on the average strength of both real and imaginary components. A first remarkable attempt has been performed by Siska with the adoption of the Tang-Toennies (TT) potential model for ion-atom interaction (Tang and Toennies, 1984; Siska, 1986). The same procedure has been extended by Ohno et al. to describe the radial dependence of the interaction in the exit channel of auto-ionization promoted by He^*^(^1^S, ^3^S)-Ar collisions and assuming the anisotropy in the exit channel according to the criteria proposed by Morgner and coworkers (Hoffmann and Morgner, 1979; Ohno et al., 1996). The adopted methodology allowed these authors to obtain the branching ratio for the two Ar^+^(^2^P_3/2_) and Ar^+^(^2^P_1/2_) exit channels. Furthermore, Ohno et al. demonstrated that the stereodynamics in the collisional auto-ionization processes critically depends on anisotropic characteristics of the interaction potentials of either X^*^ + M and X + M^+^ entrance and exit channels, respectively. For such a purpose they developed a collision-energy/electron-energy resolved two-dimensional Penning Ionization Electron Spectroscopy (2D-PIES) technique (Ohno et al., 1996). In particular, stimulated by the 2D-PIES of He^*^(2 ^3^S)-N_2_, CO, and CH_3_CN systems, Ohno and coworkers performed reliable *ab initio* calculations for both anisotropic entrance and exit potentials as well as for ionization probabilities (Yamazaki et al., 2002). After that, a series of important papers were published by Ohno group on this direction (Yamazaki et al., 2002, 2005, 2007; Ohno, 2004; Khishimoto and Ohno, 2007).

More recently, in our laboratory we are working to develop a more general exhaustive approach able to describe in an internally consistent way both the entrance (X^*^ + M) and exit channels (X + M^+^), where either the real V_*t*_ and the imaginary Γ parts of the optical potential [see Equation (1)] are both anisotropic and interdependent on the selective charge transfer (CT) component triggering the process. This is a new and original approach including the reactions induced by He^*^ collisions as a particular case of more general phenomena. The basic novelty consists on the following two main points: (i) The radial dependence of the real part of the potential [V_*t*_ in the Equation (1)], both in the entrance and exit channels, is represented by an Improved Lennard Jones Potential model (ILJ) defined by few parameters related to basic physical properties of the interacting particles (Pirani et al., 2008). Its reliability, tested on high resolution scattering and spectroscopic experiments as well as by *ab initio* calculations, has been found to be comparable and even better with respect to that of multiparameter functions, widely used in the past, as the TT model (Falcinelli et al., 2017); (ii) The description of the anisotropy in both channels in terms of the same components related to the selective dependence of CT on the half-filled P orbital alignment within the intermediate collision complex; (iii) The internally consistent representation of both real V_*t*_ and imaginary Γ parts of the optical potential [see Equation (1)] which, for the first time, has been considered not independent (as it has been done until now) but interdependent being related to the selectivity of CT. Other relevant works in the stereodynamical investigation of auto-ionization processes have been carried out by Ohno and coworkers on the He^*^-molecule systems where the He^*^ is an isotropic reagent and the dependence on the features of the involved molecular orbitals has been characterized (Ohno, 2004; Yamazaki et al., 2005, 2007; Horio et al., 2006; Khishimoto and Ohno, 2007). Important contributions have been also provided by the Kasai group on the dependence of reactions on the orientation of symmetric top molecules investigated in detail considering Ar^*^ as an isotropic collisional partner (Yamato et al., 2000; Brunetti et al., 2001; Ohoyama et al., 2001).

More recently, significant investigations were performed by Narevicius and coworkers (Henson et al., 2012; Pawlak et al., 2017; Bibelnik et al., 2019) and by Osterwalder and coworkers (Jankunas et al., 2014; Gordon et al., 2017; Zou et al., 2018), studying Penning ionization processes in the sub-thermal collision energy regime by merged-beams technique. These authors pointed out important stereodynamical implications in Ne^*^+ND_3_ system (Gordon and Osterwalder, 2019), quantum state controlled cross sections for Penning and associative ionization in Ne^*^(^3^P_2_)-Ar collisions (Gordon et al., 2018), and the observation of orbiting resonances in the He^*^-Ar and H_2_ Penning ionization reactions (Henson et al., 2012).

Therefore, important questions still remain opened: they concern the modulation of the reaction probability by the relative orientation of atomic/molecular orbitals more directly involved in the processes. A correct approach should assume quantized spatial orientations along the intermolecular electric field direction that selectively correlate with specific configurations of the collision complex, the only ones effective in leading to final products.

Target of this paper is to report on recent our advances on the characterization, for some prototypical systems, of radial and orientation dependences of both real and imaginary components of the optical potential. In particular, molecular beam experiments have been performed, measuring the energy dependence of total and partial (for the formation of product ions in specific states) ionization cross sections [σ(E)] and of energy spectra of emitted electrons. In particular, being the reaction probability highest at the closest approach of the colliding partners, Penning Ionization Electron Spectra (PIESs) can be considered as a sort of “transition state spectroscopy” (Hotop et al., 1979; Morgner, 1985; Benz and Morgner, 1986).

The analysis of experiments, performed with Ne^*^(^3^P_J_) atoms and hydrogenated molecules, like water and ammonia (Ben Arfa et al., 1999; Brunetti et al., 2013; Falcinelli et al., 2016a,b, 2017), provided the dependence of the optical potential of the systems on the separation distance and on the orientation of the molecular orbitals interested in the ionization (see Figure 1C). However, these results did not include any dependence on the atomic orbital orientation/alignment, since they were referred to a statistical average over all the atomic sublevels of open-shell Ne^*^. The dependence on the atomic orbital orientation, which is quantized and masked by spin-orbit electronic couplings within the open-atom structure of reagents and products, cannot considered a trivial question.

Our recent investigations focused on the energy analysis of electrons emitted in collisions between Ne^*^(^3^P_J_) and Kr and Xe atoms under controlled kinetic energy conditions (Falcinelli et al., 2018a,b). The contributions of the different fine structure sublevels, both in the entrance and exit channels, have been separated and their collision energy dependence resolved. The analysis of the experimental findings permitted us to extract direct information on the role of the atomic anisotropy within the collision complex, determining relevant details of both real and imaginary components of the interaction. In particular, it has been emphasized that electronic rearrangements control *adiabatic* and *non-adiabatic effects* both in the entrance and exit channels and, mostly, in the transition state. It has been found that, while adiabatic effects influence essentially the anisotropy of the real part of the potential, *non-adiabatic effects* mostly control the imaginary part (Falcinelli et al., 2018a,b). However, their contributions must be not fully independent since they are simultaneously related to: (a) external electronic orbital polarization, (b) changes in the electronic angular momentum couplings and (c) selectivity of CT contributions.

The combination of all this information, representing a substantial advancement with respect to the previous works, has been here adopted to provide for the first time, on our knowledge, the state-to-state reaction probability of the system Ne^*^(^3^P_J_)-Kr.

In the following, we start from a summary of the main results obtained on the study of orientation effects of the H_2_O molecular orbitals, involved in the collisional ionization by Ne^*^(^3^P_J_) atoms and then we discuss the Ne^*^(^3^P_J_)-Kr case with the determination of the Γ components associated to the state to state processes, i.e., Ne^*^(^3^P_0_)—Kr^+^(^2^P_3/2_), Ne^*^(^3^P_0_)—Kr^+^(^2^P_1/2_), Ne^*^(^3^P_2_)—Kr^+^(^2^P_3/2_), and Ne^*^(^3^P_2_)—Kr^+^(^2^P_1/2_), including all the multiplicity of states in entrance and exit channels (Falcinelli et al., 2016a,b).

## The Ne^*^-Molecule Case

A wide literature is available on Penning ionization studies involving He^*^ and Ne^*^ metastable atoms as ionizing agents of simple hydrogenated molecules, as water (Yee et al., 1976; Cermák and Yencha, 1977; Sanders and Muschlitz, 1977; Ohno et al., 1983; Haug et al., 1985; Mitsuke et al., 1989; Ishida, 1996).

**Figure 2:**
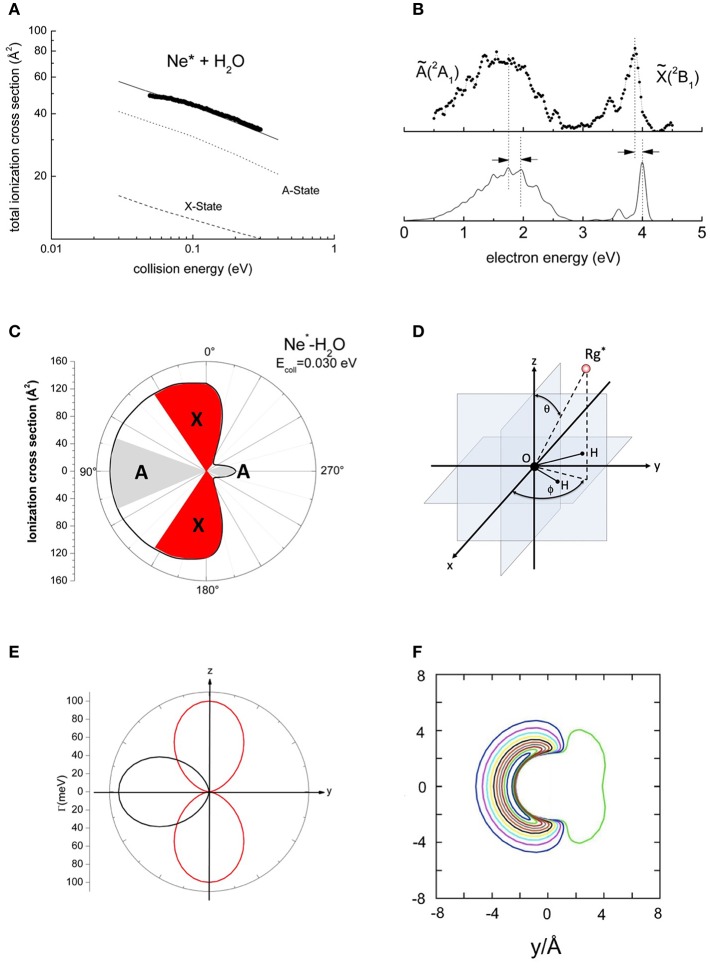
The results about the ionization of the water molecule by metastable Ne^*^(^3^P_2, 0_). **(A)** Total ionization cross sections where the contribution for the two ionic state are separated. **(B)** Penning ionization electron spectrum where the shifts with respect the nominal expected positions of the peaks are also indicated. **(C)** A polar map of the ionization efficiency in a plane perpendicular to the molecule. **(D)** The polar coordinate framework used to analyze the experimental data. **(E)** The polar plot of the imaginary component of the optical potential for the production of A (black) and X (red) states of the H_2_O^+^ product ion. **(F)** A contour map of the real part of the optical potential on the molecular plane (for details see Falcinelli et al., 2016a,b).

Collisions between Ne^*^ and H_2_O have been investigated in detail also by our group and Figure 2A reports measured absolute total and partial ionization cross sections for the formation of [H_2_O(*X* or *A*)]^+^ product ions, as a function of the collision energy (Balucani et al., 2012; Brunetti et al., 2012). They exhibit different values but similar energy dependence. The branching ratio (BR) was estimated by the areas of PIES peaks, also reported in Figure 2B, related to the formation of H_2_O^+^ in the ground *X* respect to the first excited *A* electronic state (Balucani et al., 2012; Brunetti et al., 2012; Falcinelli et al., 2016a,b). On this ground, BR is found to be of about 0.3. A similar procedure has been adopted to obtain BR for other hydrogenated molecules (Falcinelli et al., 2015b, 2016b).

In the analysis of experimental data, the Γ component of the optical potential that depends on the overlap integral between orbitals exchanging the electron has been represented by an exponential decreasing function of *R*. Its angular dependence, modulated by trigonometric functions, has been derived from Legendre polynomials (or spherical harmonics) (Falcinelli et al., 2016b). In the adopted Γ formulation both dependences of the overlap integrals on *R* and on the shape of involved molecular orbitals have been enclosed. According to the guidelines described in Falcinelli et al. (2016b), we have assumed:

(2) $$\Gamma \left(R,\;\theta ,\;\phi \right)=\;Ae^{-bR}cos^{2}\theta$$

for the removal of one electron from the almost pure *p* orbital, that is perpendicularly to the molecular plane of H_2_O, with subsequent formation of the ground *X* electronic state of ionic molecular product. In order to describe the electron ejection from the molecular orbital with cylindrical symmetry, aligned along the C_2*v*_ axis of H_2_O, which determines the formation of H_2_O^+^(*A*), the following formulation has been adopted (Falcinelli et al., 2016b):

(3) $$\Gamma \left(R,\;\theta ,\;\phi \right)=\;Ae^{-bR}sin^{2}\theta sin^{2}\phi$$

The Equation (2) holds only for 180° ≤ ϕ ≤ 360°, and outside such a range Γ becomes zero. The polar coordinate system is defined as illustrated in Figure 2D.

During the analysis, the strength of the binding energies, predicted by the real part of the optical potential for the most relevant limiting configurations of the Ne^*^-H_2_O collision complex (Brunetti et al., 2013; Falcinelli et al., 2016a) (see also Figure 2F), has been tested on some features of PIES, including also the shift respect to the peak positions measured in pure photo-ionization events by Ne(I) photons. In addition, only the *A* and *b* parameters of Γ components have been varied in order to reproduce the absolute value of total and partial ionization cross sections and their energy dependence (Falcinelli et al., 2016b). The angular dependence of the Γ components, defined by Equations (2) and (3) for three different distances chosen in the range of the classical turning points mainly probed in the investigated collision energy range, are also given in Figure 2E.

Furthermore, the combined characterization of imaginary and real parts of the optical potential permitted us to evaluate, at each collision energy, the value of partial ionization cross sections for the formation of product ions in different electronic states as a function of the polar angles (Falcinelli et al., 2016b). The obtained results emphasized the selectivity in the formation of the product ions in the different electronic states and provided crucial information on the stereo-dynamics of the auto-ionization reactions involving water, that is probably the most important prototype of hydrogenated molecules. In particular, this investigation clearly indicated that the reactions occur only for specific orientations of water molecule with respect to the approaching Ne^*^ atom. The convergence of calculated and measured cross sections and the reproduction of the BR, as deduced from the ratio of measured peaks in PIES, allowed us to estimate also the “effective” angular cones of approach where the reaction has the highest probability to occur, whose shapes are depicted in Figure 2C (Falcinelli et al., 2016a,b).

In spite of the clarification of these stereo-dynamical effects for atom-molecule collisions, a more detailed treatment should consider that the inner shell ionic core of the Ne^*^ atom exhibits a half filled *p* orbital and this exclusively promotes the reaction. Therefore, the lacking of its alignment along the *R* direction hinders the reactivity, and then in the simulations angular cone acceptances must widen in order to provide expected cross section values.

The characterization of the selective role of half-filled atomic orbitals has been the target of our most recent research (Falcinelli et al., 2018a,b), focused on metastable atom-atom systems, which represent the simplest case of stereo-selective chemical reactions. For such systems, it has been possible to investigate in detail the role of inner half-filled orbital and of its spatial quantized orientations.

## The Ne^*^-Atom Case

As mentioned above, previous studies on atom-atom and simple atom-molecule systems (Hotop and Niehaus, 1968; Hotop et al., 1971; West et al., 1975; Gregor and Siska, 1981; van den Berg et al., 1987; Aguilar Navarro et al., 1992; Brunetti et al., 1993), carried out through the measurement of energy dependence of ionization cross sections, provided only estimates of the two terms, the real V_*t*_ and the imaginary Γ parts, of the optical potential [see Equation (1)] in their isotropic radial dependence. Recent more advanced experiments with state selected Ne^*^ beams (Gordon et al., 2017; Zou et al., 2018) provided the collision energy dependence of associative to Penning branching ratio for individual sublevels of Ne^*^(^3^P_2_), an important observable, due to its expected dependence on the spatial quantization of open-shell structures within the collision complex.

Following previous pioneering experiments (Tang et al., 1972; West et al., 1975; Martin et al., 1978; Neynaber and Tang, 1979a,b), we have measured and analyzed important features of PIES spectra (Brunetti et al., 2006; Falcinelli et al., 2018a,b), in Ne^*^- Kr, Xe experiments, providing new and complementary information on the role of *adiabatic* and *non-adiabatic* electronic effects controlling structure and reactivity of the intermediate-transition state of the systems.

**Figure 3:**
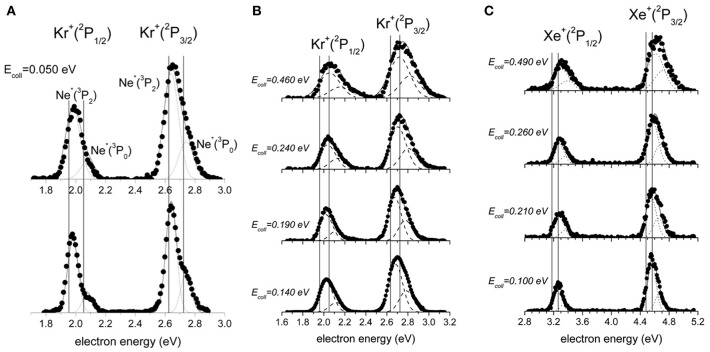
The electron energy spectra for the ionization of Kr and Xe by metastable Ne^*^(^3^P_2, 0_). **(A)** The spectrum for the ionization of Kr at 0.050 eV collision energy under low (upper part) and high (lower part) resolution conditions. **(B)** Spectra for Ne^*^(^3^P_2, 0_)-Kr ionization measured as a function of the collision energy. **(C)** Spectra for Ne^*^(^3^P_2, 0_)-Xe ionization measured as a function of the collision energy. The vertical lines define the position of peaks in canonical photo-ionization spectra.

Obtained PIESs for Ne^*^-Kr, Xe, as a function of collision energy, are reported in Figure 3, where all peaks are shown to be globally shifted respect to the canonical Ne(I) photo-ionization spectra. The shift is known to originate by the effective real potential that controls energy and structure of the collision complex. In particular, the electron emission mostly occurs from the collision complex in proximity of the closest intermolecular distance, where the time spent by the system is longer, the interaction stronger, and the reaction probability is highest. Therefore, the shift is directly related to the stability of the collision complex in the achieved configuration, depending on the different orientations permitted to the half-filled orbital of the metastable atom and on the critical balance of attractive and repulsive interactions involved.

As reported in recent papers (Falcinelli et al., 2018a,b), the combined analysis of measured PIES spectra, plotted in Figure 3, suggested the occurrence of contributions from four different reaction channels, assigned to two different spin-orbit states of the Ne^*^(^3^P_2, 0_) reagent and two different spin-orbit states of the ionic M^+^(^2^P_3/2, 1/2_) products (M = Kr, Xe). The relative probability of these four channels has been extracted by fitting the PIES spectra with four different Gaussian functions to which we have imposed the following two conditions: (i) all used functions have the same width; (ii) their relative peak position is shifted taking into account for the spin-orbit spacing of Ne^*^. The relative probability of the four channels has been then related to the relative height of the four peaks of the spectra.

The obtained results emphasized the reactivity change of ^3^P_0_ atoms with respect to ^3^P_2_, in producing ions in ^2^P_3/2_ and ^2^P_1/2_ sublevels, as a function of the collision energy. These findings have been assumed to arise from a critical balance of *adiabatic* and *non-adiabatic effects* that control formation and electronic rearrangement of the collision complex, respectively.

While the anisotropy of the real part of *W* [see Equation (1)] depends on the effectiveness of *adiabatic effects*, strength and selectivity of the imaginary component are more directly affected by *non-adiabatic effects*. The latter, however, cannot be considered fully independent on the *adiabatic ones*, since all effects are simultaneously affected by relevant changes in the electronic configurations probed by the system.

The peak positions of PIES spectra, with respect to those from Ne(I) photoionization, and their shifts with the increase of the collision energy, are found to arise from different critical balances between attractive and repulsive components of the real potential. Therefore, in the entrance channels the potential formulation must take into account that the long range interaction is confined in the non-covalent neutral atom-neutral atom type, depending on the spherical *electronic polarizability* of both atoms, where that of Ne^*^ is the highest one being determined by the outer weakly bound electron in the *3s* orbital. In the range of intermediate and short distances we have the gradual/adiabatic formation of the complex [(Ne-M)^+^]^e^*, where the open shell (Ne-M)^+^ ionic adduct is surrounded by the floppy cloud of the outer electron in excited Rydberg states. We can define a first *critical* distance, *R*_0_, being the internuclear separation where the two configurations have the same importance and consequently an intermediate situation takes place. The *R*_0_ value must be almost the same for Ne^*^-Ar, Kr, Xe systems, since they are primarily affected by the larger size Ne^*^ metastable atom (or by its polarizability), whereas it should increase when atoms with higher polarizability are involved, as is the case of He^*^(${}^{1}S_{0}$).

In order to cast light on the contribution of *adiabatic* and *non-adiabatic effects*, exploiting the analysis of the experimental observables, it has been fundamental to evaluate preliminary all relevant features of effective interaction potentials controlling the molecular dynamics both in the entrance and in exit channels. For their proper formulation, whose details are given in Falcinelli et al. (2018a,b), we have exploited a *phenomenological-semiempirical* method developed in our laboratory that combines information from theoretical approaches, useful to describe the collision events of open shell *P-state* atoms with closed shell partners, with results of scattering experiments performed with atomic beams selected in their spin-orbit sublevels *J* (Aquilanti et al., 1982, 1988, 1989, 1997; Pirani et al., 2000).

The rationalization of all experimental and theoretical findings suggested us (Falcinelli et al., 2018a,b) some crucial characteristics of *adiabatic* and *non-adiabatic effects*, summarized as it follows. *Adiabatic effects* determine:

1) The gradual transition of the interaction from the neutral atom-neutral atom representation, at long range, to the ion-neutral one surrounded by an excited electron, at short range;
2) The change in the electronic angular momentum coupling scheme, as the separation distance decreases, with the proper correlation between atomic |J,Ω >, where Ω is the absolute projection of **J** along **R**, and molecular states emerging at short distances, here represented by ^2S+1^Λ or |Λ,Ω > notations. Note that only molecular states of Σ (Λ = 0) and Π (Λ = 1) symmetry (or character) are permitted to *P-state* atomic species (Aquilanti et al., 1989) and they correspond to two different quantized spatial orientations of the half-filled *P* atom orbital respect to **R**;
3) The gradual formation of quantum molecular states with a defined symmetry from each atomic sublevel, as R decreases;
4) The dependence of the Σ and Π molecular character degree of each quantum state on the separation distance and their mixing by the spin-orbit coupling;
5) The selective bond stabilization by CT of quantum molecular states of defined symmetry, arising from the configuration interaction between entrance and exit channel states differing for one electron exchange, that represents for such systems the main contribution to the interaction anisotropy at short range (Pirani et al., 2000).

Non-adiabatic effects relate to:

1) The fast/instantaneous deformation of the floppy cloud of the most external electron, which the highest probability of occurring around *R*_0_;
2) The coupling between entrance and exit channels by CT, whose efficiency depends on *R* and on the symmetry of the molecular states coupled;
3) The mixing of the molecular states of different symmetry by the spin- orbit coupling;
4) The critical R regions where an *incipit* J decoupling occurs, proving a sort of *confusion* in the electronic angular momentum coupling scheme;
5) The contribution of the electronic-orbital coupling arising from centrifugal or Coriolis effects.

The simultaneous consideration of all these features suggested (Falcinelli et al., 2018a,b) that the electronic rearrangement triggering the transition from entrance to exit channels can involve two different complementary mechanisms. They are the following:

i) A *direct mechanism*. It is stimulated by nuclear vibration-electronic orbital motion couplings. It causes a *homogeneous electron exchange*, involving two coupling terms, pointed out as *A*_Σ−Σ_ and as *A*_Π−Π_ on the basis of the symmetry of either initial and final states of the system involved in the electron exchange. Such mechanism is expected to be more efficient at short distances where the molecular character is more defined and/or the quantized spatial orientations of the half-filled orbitals are more effective. A value of 5 has been used for the *A*_Σ−Σ_/*A*_Π−Π_ ratio, following the suggestion of Krauss (1977) analyzing the different overlap integral, for the same value of R, between atomic orbitals giving molecular states of Σ and Π character that are implicated in the CT.
ii) An *indirect mechanism*, supporting a *heterogeneous electron exchange*. Such a mechanism induces a mixing of Σ and Π character and is promoted by the spin-orbit interaction as well as by the nuclear rotation-electronic orbital motion (also called as Coriolis coupling). It includes also possible contributions from cloud rearrangements of the outer excited electron, taking into account of radiative effects in previous developed treatments (Miller and Morgner, 1977; Gregor and Siska, 1981). Two possible coupling terms, *A*_Σ−Π_ and *A*_Π−Σ_, identified on the basis of the symmetry of the initial and final states are involved in this second mechanism. Also in this case, for the same reasons discussed above in the *direct mechanism*, the *A*_Σ−Π_/*A*_Π−Σ_ ratio has been fixed equal to 5.

The peak ratio dependence on the collision energy allowed us to characterize the relative role of the two mechanisms, defined as *A*_Σ−Π_/*A*_Σ−Σ_ ratio (Falcinelli et al., 2018a,b). Moreover, the above considerations on the role of adiabatic and *non-adiabatic* effects suggest that *A*_Σ−Σ_, and consequently *A*_Π−Π_, must be rather proportional to the bond stabilization contribution by CT (Aquilanti et al., 1997; Falcinelli et al., 2017). Such contribution, that arises from the configuration interaction between quantum states of ionic adducts, *having the same symmetry and associated to entrance and exit channels*, must be also crucial in promoting the reactivity. Moreover, the proportionality is expected to depend mostly on the overlap integral between wave functions describing the ejected electron in the initial-excited and in the final-continuum state (Gregor and Siska, 1981). Since here the involved orbitals are rather diffuse, such proportionality can be assumed constant, that is independent of *R* (no radial dependence), at least in range of distances of interest. The value of the constant is expected to depend essentially on the characteristic of the electron wave function in initial excited state.

Assuming a proportionality constant equal to ½ for all processes promoted by Ne^*^(^3^P_2, 0_), from the knowledge of the bond stabilization by CT, we have derived a simple relation providing the radial dependence of *A*_Σ−Σ_. In the case of Ne^*^-Kr system (the treatment can be easily extended to other Ne^*^-Noble gas cases) it has been assumed the form

(4) $$A_{\Sigma }{{}_{-}}_{\Sigma }(meV)=\;3.35\times 10^{6}\;e^{-4.32R}$$

where *R* is given in Å.

From the collision energy dependence of the *A*_Σ−Π_/*A*_Σ−Σ_ ratio, we estimated also the following less pronounced, radial dependence for *A*_Σ−Π_

(5) $$A_{\Sigma }{{}_{-}}_{\Pi }\left(meV\right)=\;335\;e^{-1.40R}$$

where *R* is again in Å.

The additional *A*_Π−Π_ and *A*_Π−Σ_ couplings have been so indirectly characterized according to the criteria discussed above. The Γ components, depending on the symmetry of the states involved, must correspond to the so obtained coupling terms.

## Discussion and Conclusions

Our investigation suggests a tentative comparison and a combined discussion of the most important findings presented in the previous sections for both the different type of prototype systems. The results for metastable atom-hydrogenated molecule systems, concerning the selective formation of product ions in the different electronic states (see Figure 2), provided crucial information on the stereo-dynamics of these important auto-ionization reactions, especially emphasizing the dependence of the reaction probability on the molecular orientation. The adopted treatment allowed also to estimate of the magnitude order of angular cones where the processes are mainly confined. Such cones amount to about 1–2 sr, corresponding to an aperture of about 30–60 degrees. Note that the results reported in a previous paper (Falcinelli et al., 2016b) are relative values and they must be multiplied for 4π to give absolute angular cones in radiants. The so obtained cones must be taken as *effective*, since their values are the result of an average over the investigated energy range. Furthermore, *natural* molecular orientation effects should reduce the angular cone of acceptance around the direction of the most favorable configurations: they can become operative during the collision of polar molecules, as for example H_2_O, and are induced by electric field gradients in strongly anisotropic intermolecular potentials. These effects should increase when the collision energy decreases, especially in the sub-thermal energy range (Chang et al., 2013; Jankunas et al., 2014; Cernuto et al., 2017, 2018).

**Figure 4:**
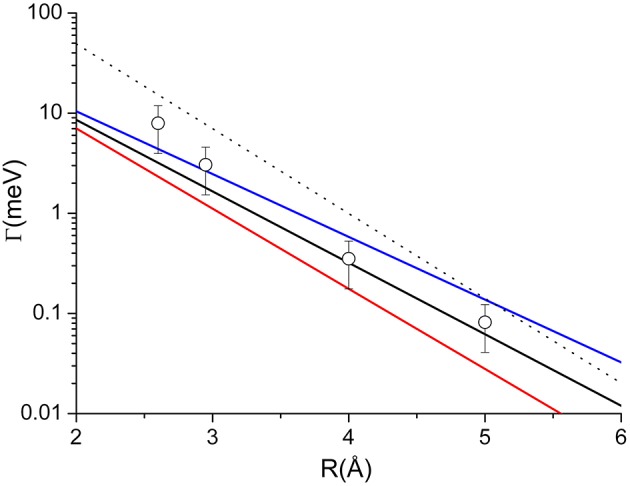
Comparison of the radial dependence of the average Γ value obtained for two prototype systems: *Dashed line*—the Ne^*^-H_2_O case, discussed in the text and giving the ground electronic state of water ion; *Full* lines—the Ne^*^-Kr case, obtained by Gregor and Siska (1981) where black, red, and blue colors represent average value, lower and upper limits, respectively. For the same system the circles are the results of the present investigation.

Adopting the potential parameters reported in Falcinelli et al. (2016b) it becomes possible to determine both the highest value of the strength of Γ, associated to the most reactive configuration, and its value estimated by averaging over the full space of the relative configurations, where the most relevant contribution comes from the angular cone where the formation of a final ionic product in a specific state is favored. Considering an *R* value of 3Å, the highest Γ value that controls the formation of water ion both in the ground and in excited electronic state, amounts to about 70 meV, respectively, while the value averaged over the complete range of configurations reduces to about 7 and 4 meV, respectively. Similar estimates apply to other hydrogenated molecules as ammonia and hydrogen sulfide (Falcinelli et al., 2014, 2016a,b). For some comparisons with results presented below, the radial dependence of the average component giving the ground state of water ion is plotted in Figure 4.

**Figure 5:**
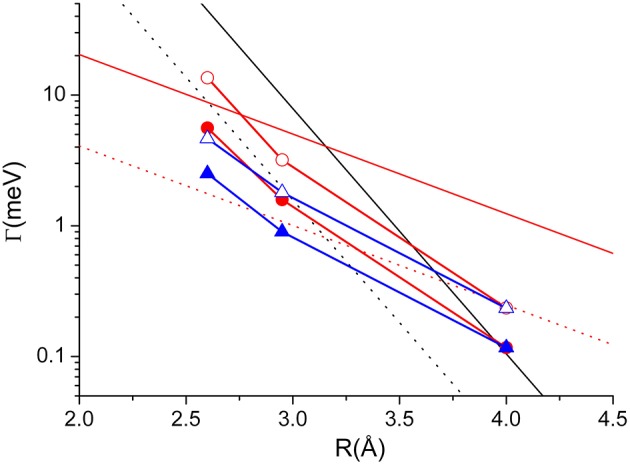
The A_Σ−Σ_(R) (full black) A_Π−Π_(R) (dashed black), A_Σ−Π_(R) (full red) and A_Π−Σ_(R) (dashed red) coupling terms determining *direct and indirect mechanism*s in auto-ionization reactions of Ne^*^-Kr system. Open and closed red circles represent the Γ components associated to ^3^P_0_-^2^P_3/2_ and ^3^P_0_-^2^P_1/2_ state to state processes; Open and closed blue triangles represent the Γ components associated to ^3^P_2_-^2^P_3/2_ and ^3^P_2_-^2^P_1/2_ state to state processes. Note that in the exit channels the reaction probability is weighted on the different degeneracy of J = 3/2 and J = 1/2 states of the ionic product.

For atom-atom cases, the Γ components, identified here with the A_Σ−Σ_(R), A_Π−Π_(R), A_Σ−Π_(R), and A_Π−Σ_(R) coupling terms, obtained according to the method summarized in the previous section, are plotted in Figure 5. Such components suggest that the dependence of the *indirect mechanism* by the internuclear distance *R* appears to be much less evident than the case of the *direct mechanism*. For this reason, the *indirect mechanism* becomes less relevant when the collision energy increases (or when the probed separation distance decreases), since the *direct mechanism* is favored by the more pronounced molecular character attained by the initial states.

The Γ components, evaluated in previous studies and showing only a radial dependence (Gregor and Siska, 1981), must considered as effective-average radial terms. An important test of our methodology can be achieved by performing a weighted sum of the obtained coupling terms by taking into account for the statistical populations of ^3^P_2_ and ^3^P_0_ fine levels of Ne^*^ reagent, the degeneracy of the different quantum states accessible to the colliding system and the dependences of the Σ and Π character of each state on *R*. The average Γ values so obtained are reported in Figure 4, where they are compared with those of Ne^*^-water, to emphasize that the strength is in the same scale, and with the empirical function obtained by Gregor and Siska (1981) for the same system. The very good agreement obtained authorized us to attempt a de-convolution of Γ in order to determine the state-to state Γ components. The obtained results, plotted in Figure 5, show a well evident dependence of the reaction probability on the fine level of both reagents, including ^3^P_0_, and products.

It should be emphasized that the methodology proposed by us allows to characterize both the real and the imaginary parts of the optical potential within the same internally consistent framework, that takes into account properly all the features of *P* atom interaction and of its collision dynamics. Next steps concern the extension of the methodology to other atom-atom systems and to the obtaining the dependence of Γ on both *J* and Ω quantum numbers of reagent and products, which is of interest for the investigation of quantum effects in the coherent control of collision processes, promoting both Penning and associative ionization, from under ultra-cold up to thermal conditions (Arango et al., 2006a,b).

Moreover, our investigation on atom–atom auto-ionization reactions, takes into account for the first time in the PIES analysis of experimental spectra both decoupling schemes of the electronic angular momentum and the Coriolis coupling effects, providing new insights on the microscopic electron rearrangements within the transition state of auto-ionization reactions. In particular, both real and imaginary parts of the optical potential [see Equation (1)] have been reported in an internally consistent way being related to basic selectivities of CT. On this ground the electronic rearrangements have been classified as *adiabatic* and *non-adiabatic effects*: they are controlled by the anisotropy of specific interaction components and consequently their characterization is of relevance to rationalize also the behavior of atom-molecule systems. In particular, it has to be noted that orientation effects of polar molecules can spontaneously takes place, making accessible only some specific geometries of the transition state. This can occur at low collision energies, where *non-adiabatic* contributions tend to vanish, and under these conditions the reaction probability should be almost independent on the collision energy (Jankunas et al., 2014).

In conclusion, we proposed a new and original method to fully describe, and including in a more general picture, the stereo-dynamics of the state to state auto-ionization reactions. For such a purpose we exploited the ample phenomenology achieved in the last 30 years by our group on the selective collision dynamics of open-shell atoms. Furthermore, our model appears to be able also to clarify in detail the role of electronic rearrangements within the transition state of many other types of chemical processes, that are more difficult to characterize.

## Author Contributions

All authors planned experiments and made discussion about the results. SF and PC managed the experiments. SF, FV, and FP analyzed the results. All authors participated in writing the manuscript.

### Conflict of Interest Statement

The authors declare that the research was conducted in the absence of any commercial or financial relationships that could be construed as a potential conflict of interest.
